# Understanding Why Tourists Who Share Travel Photos Online Give More Positive Tourism Product Evaluation: Evidence From Chinese Tourists

**DOI:** 10.3389/fpsyg.2022.838176

**Published:** 2022-04-08

**Authors:** Xiuyuan Tang, Yanping Gong, Chunyan Chen, Suying Wang, Pengfei Chen

**Affiliations:** ^1^School of Business, Central South University, Changsha, China; ^2^School of Business, Central South University of Forestry and Technology, Changsha, China

**Keywords:** photo-sharing behavior, pleasure, social experience, self-construal type, tourism product evaluation

## Abstract

This study tested a conceptual model in which photo-sharing behavior during travel elicits tourists’ emotional state, and in turn improves evaluation of the tourism product. The research results in the context of tourist attractions and restaurants provide support for the proposed model. Specifically, tourists’ photo-sharing behavior was significantly associated with more positive product evaluation, both directly and indirectly *via* the emotion of pleasure. These associations were stronger when the interdependent self-construers had good social experience. The results provide practical guidance for marketers to developing marketing strategy.

## Introduction

With the widespread use of the Internet and social media, it has become common to share travel experiences by posting photos online (Jansson, 2018). The literature on photo-sharing behavior mainly focuses on the antecedents and meanings of consumers’ photo-sharing behavior (Chae et al., 2017; Taylor, 2020). Previous studies have found that among younger consumers, the main purpose of taking photos is not to preserve travel memories, but to be able to share the photos with a friendship circle (Grewal et al., 2019). People also express their identities by allowing selective exposure to photos (Barasch et al., 2017b). Such sharing is not only free advertising to those who view the photos, but it also increases the possibility that information about the product experience will spread by word of mouth (Godes and Mayzlin, 2004; Chae et al., 2017).

Social network sharing behavior will have an impact on consumers’ psychology and behavior. Previous literature on social network sharing mainly discusses the role of sharing behavior on the information recipients’ response, *via* word-of-mouth communication (Lien and Cao, 2014), user-generated content and product reviews in stimulating sales (Zhang et al., 2017). In recent years, some scholars have paid attention to the influence of sharing behavior on the sharers themselves. These studies have found that sharing on social networks not only affects consumers’ subjective well-being (Lee et al., 2010; Duan and Dholakia, 2017), but also affects consumers’ consumption behavior by affecting consumers’ cognition and emotion (Asih et al., 2020). For example, when sharing products related to identity, consumers will be less willing to buy the same or similar products later (Grewal et al., 2019).

We focus on the increasingly common phenomenon of tourists taking photos and sharing them on social networks. On the basis of previous research, it is the first time to link tourists’ photo sharing and tourism product evaluation. This study examined tourists’ photo-sharing behavior as an influence on pleasure and subsequent evaluation of tourism product, and tested whether social experience and self-construal type associated with posting travel photos online acts as a mechanism of this influence. The results of this study can provide guidance for destination managers and marketers of tourism-related supply components (i.e., attractions, restaurants, etc.) to develop more effective marketing strategies and improve the competitiveness of tourist destinations.

## Theoretical Framework and Hypotheses

### Photo-Sharing Behavior and Product Evaluation

Tourists’ familiarity with tourism products and perception of product functions will directly affect their evaluation of tourism products (Duffett, 2015). The comfort and flexibility provided by social media encourage people to share their shopping and travel experiences more than ever before (Tamir and Mitchell, 2012; Chu et al., 2021). Over 70% of posts on social media are about self-expression or personal consumption experiences (Naaman et al., 2010). The use of social media on smartphones has further increased the immediacy of self-expression. Previous studies have shown that this kind of self-sharing could affect consumers’ attitudes (Glass, 2017), judgments (Byun et al., 2018), consumption choices (Rifkin et al., 2021), and brand preferences (Rozenkrants et al., 2017).

Photo-sharing is a very common way of user-generated content generation (Coary and Poor, 2016; Shi et al., 2018). People take photos to record their travel experiences and selectively share and publish them to express their personal identity (Barasch et al., 2017b; Krämer et al., 2017). Sharing photos of travel experiences on social networks can establish a connection between the sharers and the experience products, increase their familiarity with the experience products, and thus improve their evaluation of related tourism products (Gilovich et al., 2015; Zhu et al., 2019).

In addition, photo-sharing behavior can lead to delayed consumption. Although the traditional discounted utility theory maintains that consumers prefer to spend money earlier rather than later (Loewenstein and Prelec, 1992). There is also evidence that the expectation of future positive valence experiences leads consumers to have more positive effects (Nowlis et al., 2004). For example, photographing beautiful food during the travel briefly delays consumption, and tourists can participate and interact with the beautiful food during the delay (Coary and Poor, 2016). This short delay evokes the taste of the coming consumption experience, which can increase the functional perception of the experienced product (Nowlis et al., 2004), contributing to the improvement of product evaluation and attitude (Coary and Poor, 2016).

In summary, we propose Hypothesis 1:

*H1: Tourists’ photo-sharing behavior during travel will affect their evaluation of tourism product.*

### The Mediating Role of Pleasure

Consumer emotions are emotional reactions triggered during the consumer experience (Westbrook and Oliver, 1991). Consumers’ emotions (such as happiness or sadness) also affect their attitudes and behaviors (Creusen et al., 2018; Zablocki et al., 2019). The extant tourism literature has shown that emotions in the travel process will affect residents’ attitudes toward tourism (Ouyang et al., 2017), tourist satisfaction (Su et al., 2020b), word-of-mouth (Su et al., 2016), revisiting intention (Su et al., 2014), and destination identity (Su and Swanson, 2017). Positive emotions can have a favorable bias effect on product attitudes (Kim et al., 2010). These studies found that those experiencing positive emotions tend to have more positive attitudes toward products than those experiencing negative emotions (Howard and Gengler, 2001; Kim et al., 2010).

Active involvement in an experience can increase the feeling of pleasure (Csikszentmihalhi, 2020), a positive emotion (Su et al., 2020a) that reflects the degree to which someone is content, satisfied, and gratified (Walsh et al., 2011). Taking travel photos is a form of involvement in the consumptive experience that can increase the sense of meaning and pleasure (Lambert et al., 2013). This type of involvement or participation includes intense focus during the consumptive experience. When tourists take photos of tourist attractions during their travels, the objects they want to capture are part of the information field related to the current task; by not switching tasks, they do not suffer cognitive overload and the pleasant mood is sustained (Achtziger et al., 2008). In other words, taking a photo makes tourists look at the product they are experiencing longer and more often, increasing the sense of participation (Barasch et al., 2017a) and thus increasing pleasure.

According to the feelings-as-information theory, tourists will take their current feelings as the information source when evaluating tourism products (Schwarz, 2011). The judgment of emotional consistency reflects the influence of consumer emotions on perceived product performance (Kim et al., 2010). Pleasant will increase product evaluation, and unpleasant will reduce product evaluation (Creusen et al., 2018). Based on the above literature, we assert that photo-sharing behavior during travel will generate the emotion of pleasure, thus improving product evaluation.

In summary, we propose the following hypothesis:

*H2: Pleasure plays a mediating role in the positive influence of tourists’ photo-sharing behavior on tourism product evaluation.*

### The Moderating Role of Social Experience

Social experience is important in product design (Ponsignon and Derbaix, 2020) as it affects users’ satisfaction and willingness to continue using the product (Zhai et al., 2019). Existing studies believe that social experience elements mainly focus on social context perception and sharing interaction mechanism (Liu, 2016).

Tourists taking photos during the consumption experience will bring them closer to the product, increase their interaction with the product, stimulate tourists’ positive emotions, and produce pleasure (Guo et al., 2020). Meanwhile, posting photos is also an important way of self-expression (Young, 2011). Generally, people are willing to show their good side to others on social networks (Wilcox and Stephen, 2013). Social context affects the tourists’ participation interest and expected enjoyment (Zhu et al., 2019). In addition, interaction and feedback are key elements of social media (Felix et al., 2017).

According to symbolic interactionism, sharing photos on social networks can realize interaction with online friends through posting, commenting, likes, forwarding and other forms (Blumer, 1986; Guo et al., 2020). This kind of social interaction can strengthen the social relationship between the sharer and the online friends (Ramanathan and McGill, 2007). When there are more likes and positive comments on social platforms, people report higher levels of happiness (Grieve et al., 2013), and perceive themselves as more positive and important (Zell and Moeller, 2018). The positive emotions generated by these interactions play an important role in reducing consumers’ negative attitudes toward the target brand (Malik and Hussain, 2017; Martínez-Ruiz et al., 2017). However, when the social experience is poor (for example, few or no likes, positive comments and reposts), individuals feel nervous and psychologically uncomfortable, which may distort their evaluation of the product (Zhu et al., 2019). Therefore, we propose the following hypothesis:

*H3: Compared with the poorer social experience, the better social experience increases the relationship between pleasure and tourism product evaluation.*

### Self-Construal Type as a Moderator

People vary in how they perceive themselves in relation to others and the social environment (Markus and Kitayama, 1991). According to self-construal theory, independent self-construers value their independence, are aware of the intrinsic self attributes of others, and tend to think in an analytical way; interdependent self-construers focus on self-regard as a part of the group, maintain harmonious interpersonal relationships, adapt to others, and tend to think in a holistic way (Nisbett et al., 2001; Monga and John, 2008). This individual difference in self-construal can influence consumers’ judgment and decision-making about products (Zollo, 2021).

The distinction between analytical thinking (a characteristic of independent self-construal) and holistic thinking (a characteristic of interdependent self-construal) may be especially helpful in predicting consumer behavior (Gao et al., 2021). Previous studies have found that analytical thinkers view product and display content as separate data blocks (Zhu and Meyers-Levy, 2009). Their behavior and decision-making styles are more independent than interdependent self-construers, and they focus more on expressing the individual’s own personality and characteristics (Ybarra and Trafimow, 1998; Hellmann et al., 2021). After photo-sharing, independent self-construers treat social experience and tourism products separately, so social experience has little impact on consumers’ sense of pleasure and evaluation of tourism products.

By contrast, holistic thinkers are more likely to view the various performance of a product on a continuum with the overall impression of the product (Zhu and Meyers-Levy, 2009). Interdependent self-construers attach importance to social connection with others and consider social norms rather than personal attitudes before making decisions (Hellmann et al., 2021). Friends’ attitudes and opinions are important to them (Gao et al., 2021). Therefore, social experience plays a considerable role in the decision-making process of interdependent self-construers. Compared with independent self-construers, interdependent self-construers have a greater impact on the moderating relationship of social experience. When interdependent self-construers experience better social experiences, they report higher levels of pleasure. Higher pleasure leads to more positive evaluations of tourism products by tourists (Zhu et al., 2019).

Thus, the following hypotheses are proposed:

*H4: The three-way interaction of self-construal type, social experience and pleasure affect tourists’ evaluation of tourism products. Specifically, when tourists are interdependent self-construers and have better social experience, pleasure has the greatest positive impact on the evaluation of tourism products.*

**FIGURE 1:**
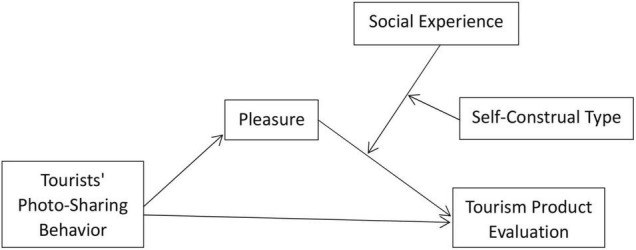
Overview of the hypothesized moderated mediation model.

We studied two typical types of travel photos, namely tourist attractions and dining. Study 1 tested H1 and H2. Study 2 replicated these results and in addition tested H3 and H4. Figure 1 shows the proposed model.

## Study 1: Photo-Sharing Behavior of Tourist Attractions and Tourism Product Evaluation

### Methods

#### Participants and Procedure

Study 1 examined the potential difference in tourism product evaluation made by tourists who did and did not share photos of tourist attractions online. The participants were recruited through the Questionnaire Star platform. Participants who were eligible to participate were at least 18 years old, active on social media and had traveled at least 60 kilometers in the last 6 months. We recruited 342 subjects through the platform, and then chose 303 participants who met these criteria. The current study was approved by the research ethics committee at the corresponding author’s institution. All participants provided written informed consent.

Participants were randomly divided into two groups (167 in the photo-sharing group, 136 in the non-photo-sharing group). Those in the experimental group were given the direction, “Please recall the last time you shared photos of a tourist attraction.” Participants in the control group were directed, “Please think about the last time you traveled but didn’t share photos of a scenic spot.” Both groups reported the pleasure score of the shared experience, and then evaluated the product. In the final sample, 54.10% were women and 45.90% were men. Self-reported age was 18-56 years old, with an average age of 23.47 years old (SD = 4.32). In terms of educational background, 30.70% had a graduate’s degree or above, 64.40% had a bachelor’s degree, 4.30% finished junior college, and 0.70% finished senior high school.

#### Measures

All English language scales were translated into Chinese using a translation and back-translation procedure. The 3-item pleasure was measured using a 7-point Likert scale adapted from Watson and Clark (1992), including “satisfied”, “pleased”, and “contented”. Tourism product evaluation was based on four 7-point semantic difference scales (bad/good, negative/positive, unpleasant/pleasant and dislike/like). Cronbach’s alpha for the pleasure scale and the tourism product evaluation scale was 0.86 and 0.86, respectively. The reliability of two scales is good.

#### Analysis Strategies

Hypothesis 1 was tested using One-way ANOVA (analysis of variance). The influence of tourists’ photo-sharing behavior on tourism product evaluation is tested in 303 sample data. H2 was tested using SPSS PROCESS Model 4. The mediating role of pleasure was examined in sample data. The bootstrap-based 95% confidence intervals with bias correction for simple effects were generated by 5,000 iterations of bootstraps.

### Results

Table 1 displays the variable means, standard deviations, and correlations for Study 1. As shown in Table 1, tourists’ photo-sharing behavior was positively correlated with tourism product evaluation (*r* = 0.34, *p* < 0.001). In addition, tourists’ photo-sharing behavior was positively correlated with pleasure (*r* = 0.36, *p* < 0.001), and pleasure was also positively associated with tourism product evaluation (*r* = 0.45, *p* < 0.001). The results preliminarily supported H1 and H2.

**TABLE 1:** Means, standard deviations, and correlations among study variables in Study 1.

Variables	*M*	*SD*	1	2	3	4	5
1. Sex	1.54	0.50					
2. Age	23.47	4.32	−0.08				
3. Education level	4.25	0.56	−0.01	−0.09			
4. Tourists’ photo-sharing behavior	0.55	0.50	0.01	0.03	0.01		
5. Pleasure	5.76	0.94	0.02	0.07	0.01	0.36***	
6. Tourism product evaluation	6.11	0.72	0.07	−0.01	0.10	0.34***	0.45***

n = 303. *p < 0.05, **p < 0.01, ***p < 0.001.

Hypothesis 1 proposed that tourists’ photo-sharing behavior during travel would affect their evaluation of tourism product. Photo-sharing behavior was used as the independent variable, with tourism product evaluation as the dependent variable. The photo-sharing group’s tourism product evaluation (M = 6.33, SD = 0.62) was significantly higher than that of the non-photo-sharing group (M = 5.84, SD = 0.73), F (1,301) = 40.47, *p* < 0.001. H1 was supported.

Hypothesis 2 predicted that pleasure would play a mediating role in the positive influence of tourists’ photo-sharing behavior on tourism product evaluation. PROCESS is an add-on package for SPSS that allows for tests of mediation, moderation, and conditional effects based on ordinary least squares or logistic regression (Hayes, 2018). The bootstrap-based 95% confidence intervals with bias correction for simple effects were generated by 5,000 iterations of bootstraps.

As shown in Table 2, the association between tourists’ photo-sharing behavior and pleasure was significant (Equation 1, B = 0.68, *p* < 0.001), and the association between pleasure and tourism product evaluation was significant (Equation 2, B = 0.12, *p* < 0.001). In addition, there was a significantly positive relationship between tourists’ photo-sharing behavior and tourism product evaluation (Equation 3, B = 0.49, *p* < 0.001). Taken together, H2 was supported.

**TABLE 2:** Regression results for mediation effect between tourists’ photo-sharing behavior and tourism product evaluation in Study 1.

Variables	Equation 1 (Pleasure)		Equation 2 (Tourism product evaluation)		Equation 3 (Tourism product evaluation)	
	*B*	*SE*	*B*	*SE*	*B*	*SE*
Control variables						
Sex	0.03	0.10	0.09	0.07	0.10	0.08
Education level	0.01	0.09	0.12	0.06	0.13	0.07
Independent variable						
Tourists’ photo-sharing behavior	0.68^***^	0.10	0.30***	0.08	0.49***	0.08
Mediator						
Pleasure			0.12***	0.04		
*R* ^2^	0.13^***^		0.25***		0.13***	

n = 303. *p < 0.05, **p < 0.01, ***p < 0.001.

## Study 2: Photo-Sharing Behavior During Meals and Tourism Product Evaluation

### Methods

#### Participants and Procedure

The purpose of Study 2 was to replicate the findings of Study 1 (pleasure mediated the association between photo-sharing behavior and tourism product evaluation) in another sample, and to examine the moderating effects of social experience and self-construal type on the mediation process. The data came from another group of participants on the Questionnaire Star platform. The manipulation procedure was similar to Study 1, in which the experimental group participants were asked to recall: “Please think about the last time you shared photos during a meal on a trip.” Participants in the control group were asked to recall: “Please think about a recent trip where you didn’t share photos during a meal.” In addition to pleasure and tourism product evaluation measures, Study 2 also collected data on social experience and self-construal type.

After screening, 320 available responses were analyzed (157 in the photo-sharing group and 163 in the non-photo-sharing group). In this study, 55.90% of participants were women and 44.10% were men. Age was directly entered by the participants on the blank form. The age range reported by the participants was 18-50 years old, with an average age of 24.08 years old (SD = 4.82). In terms of educational background, 32.80% of the participants had a graduate’s degree or above, 60% had a bachelor’s degree, 4.10% finished junior college, 2.20% finished senior high school and 0.90% finished junior high school or below.

#### Measures

The measures of pleasure and tourism product evaluation were translated for Study 1. These same translated measures were used in Study 2. In this study, the Cronbach’s alphas were 0.81 and 0.95 for pleasure and tourism product evaluation, respectively. The social experience measurement adapted from Consumption Experience Scale (Triantafillidou and Siomkos, 2014). An example item is “receiving feedback from friends makes me feel noticed.” In the current study, the Cronbach’s alpha was 0.91.

Self-construal types were measured using the Trait Self-Construal Scale (Singelis, 1994). The scale includes two subscales: independent self-construal and interdependent self-construal. There are 12 items on each subscale. Two example items are “My happiness depends on the happiness of those around me” (interdependent subscale) and “I act the same way no matter who I am with” (independent subscale). Each subscale score was calculated as the mean of the item scores. We calculated a difference score as the interdependent self-construal subscale score minus the independent self-construal subscale score. If the difference was negative, the participant was classified as an independent self-construer, denoted as 0; if the difference was positive, the participant was classified as an interdependent self-construer, denoted as 1. In the current study, the Cronbach’s alphas were 0.85 and 0.79 for interdependent self-construal and independent self-construal, respectively.

#### Analysis Strategies

Hypothesis 1, 2 were tested in the same way as in Study 1. One-way ANOVA (analysis of variance) was used to test H1 and PROCESS Model 4 was used to test H2. In Study 2 we also tested two additional hypotheses (H3 and H4). PROCESS Model 1 was used to test H3. The moderating role of social experience was examined in 320 sample data. PROCESS Model 3 was used to test H4. The three-way interaction of self-construal type, social experience and pleasure on tourists’ evaluation of tourism products was examined. Same as Study 1, the bootstrap-based 95% confidence intervals with bias correction for simple effects were generated by 5,000 iterations of bootstraps.

### Results

Table 3 displays the variable means, standard deviations, and correlations for Study 2. As shown in Table 3, tourists’ photo-sharing behavior was positively correlated with tourism product evaluation (*r* = 0.32, *p* < 0.001). In addition, tourists’ photo-sharing behavior was positively correlated with pleasure (*r* = 0.19, *p* < 0.01), and pleasure was also positively associated with tourism product evaluation (*r* = 0.30, *p* < 0.001). Thus, as in Study 1, the results preliminarily supported H1 and H2.

**TABLE 3:** Means, standard deviations, and correlations among study variables in Study 2.

Variables	*M*	*SD*	1	2	3	4	5	6	7
1. Sex	1.56	0.50							
2. Age	24.083	4.82	−0.00						
3. Education level	4.22	0.70	0.07	0.06					
4. Tourists’ photo-sharing behavior	0.50	0.50	−0.04	−0.04	0.08				
5. Pleasure	5.62	0.90	−0.03	0.03	0.02	0.19**			
6. Social experience	5.29	1.20	0.00	0.07	0.02	0.13*	0.34***		
7. Self-construal type	0.56	0.50	0.12*	−0.05	0.02	0.11	−0.01	0.06	
8. Tourism product evaluation	5.56	1.23	0.03	−0.03	0.00	0.32***	0.30***	0.19**	0.13*

n = 320. *p < 0.05, **p < 0.01, ***p < 0.001.

Findings indicated that the photo-sharing group’s tourism product evaluation (M = 5.96, SD = 0.99) was significantly higher than that of the non-photo-sharing group (M = 5.18, SD = 1.32), F (1, 318) = 35.30, *p* < 0.001. H1 was supported.

As shown in Table 4, the association between tourists’ photo-sharing behavior and pleasure was significant (Equation 4, B = 0.33, *p* < 0.001), as was the association between pleasure and tourism product evaluation (Equation 5, B = 0.35, *p* < 0.001). In addition, there was a significantly positive relationship between tourists’ photo-sharing behavior and tourism product evaluation (Equation 6, B = 0.79, *p* < 0.001). These results replicated those found in tests of H2 in Study 1. In summary, pleasure mediated the association between photo-sharing behavior and tourism product evaluation, both for photos of attractions (Study 1) and photos of meals (Study 2).

**TABLE 4:** Regression results for mediation effect between tourists’ photo-sharing behavior and tourism product evaluation in Study 2.

Variables	Equation 4 (Pleasure)		Equation 5 (Tourism product evaluation)		Equation 6 (Tourism product evaluation)	
	*B*	*SE*	*B*	*SE*	*B*	*SE*
Control variables						
Sex	−0.04	0.10	0.12	0.13	0.11	0.13
Education level	0.01	0.07	−0.05	0.09	−0.05	0.09
Independent variable						
Tourists’ photo-sharing behavior	0.33^***^	0.10	0.67***	0.13	0.79***	0.13
Mediator						
Pleasure			0.35***	0.07		
*R* ^2^	0.04^**^		0.16***		0.10***	

n = 320. *p < 0.05, **p < 0.01, ***p < 0.001.

**FIGURE 2:**
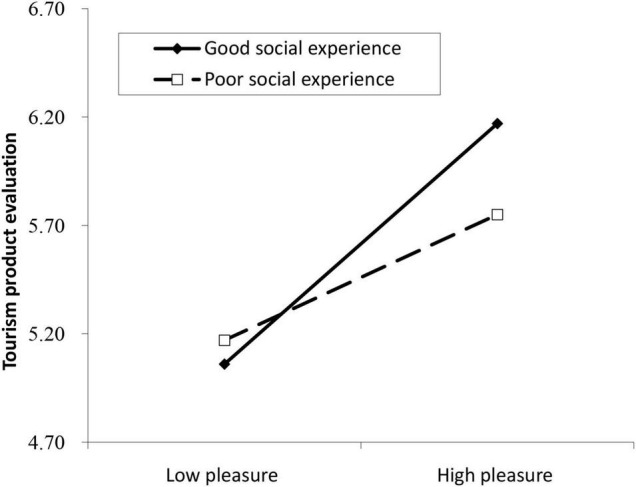
Moderating effect of social experience on the relationship between pleasure and tourism product evaluation in study 2.

In Study 2 we also tested two additional hypotheses (H3 and H4). H3 predicted that compared with the poorer social experience, the better social experience would increase the relationship between pleasure and tourism product evaluation. As shown in Table 5, the effect of the interaction between pleasure and social experience on tourism product evaluation was significant (Equation 7, B = 0.14, *p* < 0.05). The test of simple slopes (Figure 2) showed that the effect of pleasure on tourism product evaluation was larger in the context of better social experience *(*B*_*simple*_* = 0.55, *p* < 0.001, 95% CI = [0.34, 0.76]) than in the context of poorer social experience *(*B*_*simple*_* = 0.29, *p* < 0.001, 95% CI = [0.13, 0.45]). Taken together, the results supported H3.

**TABLE 5:** Regression analyses of tourists’ photo-sharing behavior predicting tourism product evaluation in Study 2.

Variables	Equation 7		Equation 8	
	*B*	*SE*	*B*	*SE*
Control variables				
Sex	0.11	0.13	0.10	0.13
Education level	−0.02	0.09	−0.02	0.09
Independent variable				
Tourists’ photo-sharing behavior				
Mediator			.	
Pleasure	0.41***	0.08	0.37***	0.08
Moderators				
Social experience	0.07	0.06	0.12	0.06
Self-construal type			0.17	0.14
Interaction terms				
Pleasure × Social experience	0.14*	0.05	0.17**	0.06
Pleasure × Self-construal type			0.51**	0.16
Social experience × Self-construal type			0.10	0.11
Pleasure × Social experience × Self-construal type			0.33** 0.11	
*R* ^2^	0.12^***^		0.19***	

n = 320. *p < 0.05, **p < 0.01, ***p < 0.001.

Hypothesis 4 predicted that the three-way interaction of self-construal type, social experience and pleasure affect tourists’ evaluation of tourism products. When the tourists were interdependent self-construers and the social experience was better, the positive impact of pleasure on tourism product evaluation was the greatest. Table 5 (Equation 8) shows that the effect of the three-way interaction among pleasure, social experience, and self-construal type on tourism product evaluation was significant (B = 0.33, *p* < 0.01).

**FIGURE 3:**
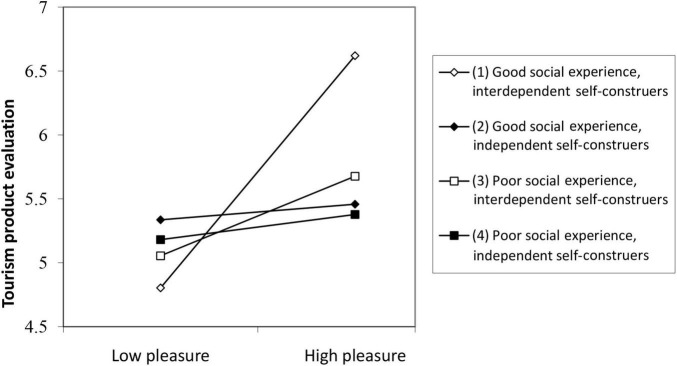
Three-way interaction of pleasure, social experience and self-construal type.

To interpret the three-way interaction (Aiken et al., 1991), the score on pleasure was divided into high and low levels, and the score on social experience was divided into good and poor levels, using the mean as the boundary between levels. Self-construal type was already a dichotomous variable. The interaction among the three dichotomous variables in predicting the continuous tourism product evaluation score is illustrated in Figure 3. Interdependent self-construers who reported high pleasure and good social experience gave the highest tourism product evaluation. In other words, the association between pleasure and tourism product evaluation, which was documented in both Study 1 and here in Study 2, was strongest among tourists who were interdependent self-construers and had a good social experience. However, for independent self-construers, the relationship between pleasure and tourism product evaluation is not affected by the quality of social experience. The results support H4.

We also used simple slope and slope difference tests (Dawson and Richter, 2006) to further analyze the three-way interaction of pleasure, social experience and self-construal type in predicting tourism product evaluation scores. These tests break the three-way interaction into two-way interactions to examine the slope of each of the two independent variables as predictors of the dependent variable. First, each slope is tested to determine if it is significantly different from zero. Second, the two slopes are compared to see if one is significantly steeper than the other—that is, if one independent variable is stronger than the other as a predictor of the dependent variable.

Consistent with H4, the results of simple slopes tests (Table 6) showed that when tourists were interdependent self-construers with good social experience, higher pleasure was associated with significantly higher tourism product evaluation (Slope 1: *t* = 3.16, *p* < 0.01). However, when tourists were independent self-construers, higher pleasure was not significantly correlated with tourism product evaluation, regardless of whether social experience was good (Slope 2: *t* = 0.17, *p* > 0.05) or poor (Slope 4: *t* = 0.32, *p* > 0.05). The results also support H4.

**TABLE 6:** Simple slope and slope difference tests for three-way interaction in Study 2.

Simple slope tests		
Slopes	Gradient of simple slope	*t*-value
1 (Good social experience, Interdependent self-construers)	0.91	3.16**
2 (Good social experience, Independent self-construers)	0.06	0.17
3 (Poor social experience, Interdependent self-construers)	0.31	1.23*
4 (Poor social experience, Independent self-construers)	0.10	0.32

**Slope difference tests**		

**Pair of slopes**	**Slope difference**	***t*-value**

1 and 2	0.86	1.37*
1 and 3	0.60	1.19*
1 and 4	0.81	4.57***
2 and 3	−0.25	−1.07*
2 and 4	−0.04	−0.06
3 and 4	0.21	0.40

n = 320. *p < 0.05, **p < 0.01, ***p < 0.001.

Further tests of differences between simple slopes showed that when tourists were interdependent self-construers, the two-way interaction between pleasure and social experience was significant (Slopes 1 and 3: *t* = 1.19; *p* < 0.05). However, when tourists were independent self-construers, the two-way interaction between pleasure and social experience was not significant (Slopes 2 and 4: *t* = −0.06; *p* > 0.05). When tourists had a good social experience, the interaction between pleasure and self-construal type was significant (Slopes 1 and 2: *t* = 1.37, *p* < 0.05). However, when the social experience of tourists was poor, the interaction between pleasure and self-construal type was not significant (Slopes 3 and 4: *t* = 0.40; *p* > 0.05). In addition, the impact of the two-way interaction between social experience and self-construal type was significant in predicting pleasure and tourism product evaluation (Slopes 1 and 4: *t* = 4.57; *p* < 0.001; Slope 2 and 3: *t* = −1.07; *p* < 0.05). These results further support H4. Both social experience and self-construal type moderated the association between pleasure and tourism product evaluation.

## Discussion

In this study, we found a positive correlation between tourists’ photo-sharing behavior and tourism product evaluation. Previous research has shown that narcissism, self-promotion and envy can motivate tourists to share travel photos on social media (Taylor, 2020), and thus react to information recipients (Lien and Cao, 2014; Zhang et al., 2017; Pop et al., 2021). Compared with previous studies, our study mainly focuses on the influence of tourists’ sharing behavior on tourists’ own psychology and behavior. Our research found that sharing photos of travel experiences on social networks can establish a connection between the sharers and the experience products, increase their familiarity with the experience products (Gilovich et al., 2015), and the functionality of the experience product perception (Nowlis et al., 2004), thereby improving sharers’ evaluation of related tourism products (Coary and Poor, 2016; Zhu et al., 2019). This study enriches the aftereffect research of photo-sharing behavior. For businesses, taking the initiative to share photos on social media can enable more potential customers to see the company and product information, and tap into a bigger market. This kind of word-of-mouth recommendation is free publicity for the company, and it is more convincing than the company’s advertising (Godes and Mayzlin, 2004; Chae et al., 2017). Given the importance to the tourism industry of encouraging tourists to share photos (Taylor, 2020), destination managers and marketers of tourism-related supply components (i.e., attractions, restaurants, etc.) should create a physical environment that inspires users to take photos and share them on social networks. For example, distinctive decoration, appropriate lighting, unique signs, eye-catching objects, etc., will not only leave a good impression on consumers, but also enhance their willingness to take photos and share them online. Tourism businesses can also place cameras from multiple angles to capture “wonderful moments” when tourists are unable to take photos.

The second, our study found that pleasure plays a mediating role in the positive effect of tourists’ photo-sharing behavior on tourism product evaluation. Social media has become a tool for shaping perceptions, feelings and experiences (Pop et al., 2021), and consumption emotion can be used as an intermediary between customer perception and subsequent behavior (Schwarz, 2011; Su et al., 2014). A large number of studies have confirmed the general influence of consumers’ cognitive behaviors and emotions on their behavioral choices, purchasing habits, and evaluation judgments (Zablocki et al., 2019; Rydell and Kucera, 2021), and studies have found that positive emotions can have a favorable biasing effect on product attitudes (Kim et al., 2010; Creusen et al., 2018). The relevant literature on the neural mechanisms underlying the decision-making process provides a more specific and multifaceted explanation for consumer psychological and behavioral responses (Drugău-Constantin, 2019; Mirică, 2019). Consistent with previous research, our study found that tourists’ photo-sharing behaviors increased tourists’ evaluation of experiential products by stimulating pleasant emotions. We believe that taking photos make tourists look at the product they are experiencing longer and more often, increasing the sense of participation (Barasch et al., 2017a) and thus increasing pleasure. While pleasure is positively associated with higher product evaluations (Creusen et al., 2018). Our results enrich the literature on the influence of consumers’ emotions on behaviors. For tourism enterprises, tourism marketers should pay attention to the influence of tourists’ emotions, strive to create new and visually pleasing tourism products, and adopt some marketing techniques that can stimulate tourists’ positive emotions.

The third, this study found a moderating effect of social experience. In the past, the only immediate communication was among co-travelers engaged in the same consumption experience, but now social media enables tourists to interact in real time with a large number of interconnected people (Zhu et al., 2019). Previous studies have explored the different impacts on consumer attitudes and behaviors in different contexts such as the sharing economy (Graessley et al., 2019; Meilhan, 2019) and the COVID-19 pandemic (Birtus and Lăzăroiu, 2021; Watson and Popescu, 2021). The widespread use of social media may also reshape consumers’ psychology and behavior, for example: Zhu et al. (2019) found that posting food photos on social media can enhance consumers’ dining experience and lead to positive evaluation of restaurants. This study presents a more complex picture of how the social experience of sharing photos interacts with pleasure to affect consumers’ evaluation of products. Sharing travel photos on social media enables real-time interaction with social friends, and this real-time interaction can significantly impact the social experience. When there are more likes and positive comments on social platforms, people report higher levels of happiness and more pleasure (Grieve et al., 2013; Zell and Moeller, 2018). The positive emotions generated by these interactions help improve product evaluations (Malik and Hussain, 2017; Martínez-Ruiz et al., 2017). This study expands the research of consumption experience theory in an environment where social media is commonly used. Therefore, tourism marketers should also pay attention to the photos posted by tourists on social media, and interact with them through posting likes, giving positive comments and forwarding their photos, so as to create a better interactive experience for the tourists.

Furthermore, previous studies have found that interdependent self-construers are more likely to be affected by other’s attitudes and evaluation than independent self-construers (Markus and Kitayama, 1991; Monga and John, 2008). Consistent with previous studies, our study found that interdependent self-construers attach more importance to social experience, and when they have a better social experience, interdependent self-construers have a higher degree of pleasure and the most positive evaluation of tourism products. Our findings enrich the evidence for the after-effects of differences in personal traits (self-construal type) on tourists’ self-expression, and help clarify the role of self-construal type in the impact of tourists’ photo-sharing behavior on tourism product evaluation. Tourism companies should be aware of the differences in the personal characteristics of tourists. By actively creating topics and tags on social platforms, tourists are encouraged to post continuously and actively interact with tourists (especially interdependent self-constructors), so as to establish long-term good relationships with tourists and improve tourism competitiveness.

## Limitation and Future Research

First, as for the research on consumption experience, we mainly studied photo-sharing behavior in the context of travel. Further research is needed to determine whether the results generalize to other consumption contexts, other fields, and service experiences (such as shopping and buying clothes, etc.).

Second, this study uses the situational questionnaire method. Experimental methods can be used in future studies to further test the causal relationship between tourists’ photo-sharing behavior and tourism product evaluation, so as to increase the robustness of research results.

Third, in real life, people may also share photos online as part of their complaints about bad travel experiences. Will this kind of photo-sharing behavior amplify tourists’ dissatisfaction? Does the social experience increase tourists’ dissatisfaction even further? In addition, this study focused on tourists’ active photo-sharing behavior, without attention to the passive photo-sharing behavior of tourists under the tourism business incentive program. For example, Taobao merchants’ praise rebate activities, restaurants require customers to post photos on social networking sites in order to get discount prices or complimentary dishes. These may be directions for future research.

## Conclusion

In the current research, two studies were conducted on photo-sharing behavior at tourist attractions and while dining to clarify the mechanism by which tourists’ photo-sharing behavior influences how they evaluate the tourism product. Our results suggest that sharing travel photos online brings pleasure, and this pleasure translates into more positive tourism product evaluation. The strongest association between pleasure and tourism product evaluation was found among tourists who had an interdependent self-construal and a good social experience through photo-sharing behavior. Most previous studies have focused on the response of photo sharing to information recipients. Our study takes a step forward by examining the effects of photo-sharing behavior on the sharers themselves. Our study provides a new perspective on social media interaction, that is, how the social experience generated by photo-sharing behavior on social networks affects the correlation between pleasure and tourism product evaluation, and also explores the role of self-construal type in these relationships. This study expands the research of consumer experience theory and self-construal theory. The results can provide guidance for tourism destination managers and marketers to develop more effective marketing strategies.

## Data Availability Statement

The raw data supporting the conclusions of this article will be made available by the authors, without undue reservation.

## Ethics Statement

The studies involving human participants were reviewed and approved by Central South University Institutional Review Board. The patients/participants provided their written informed consent to participate in this study.

## Author Contributions

XT and YG contributed to the conception and design of the study. CC and PC collected the data. XT, CC, and SW performed the statistical analysis and wrote the first draft of the manuscript. YG, XT, CC, and SW contributed to manuscript revision, read, and approved the submitted version. All authors contributed to the article and approved the submitted version.

## Conflict of Interest

The authors declare that the research was conducted in the absence of any commercial or financial relationships that could be construed as a potential conflict of interest.

## Publisher’s Note

All claims expressed in this article are solely those of the authors and do not necessarily represent those of their affiliated organizations, or those of the publisher, the editors and the reviewers. Any product that may be evaluated in this article, or claim that may be made by its manufacturer, is not guaranteed or endorsed by the publisher.

## References

[B1] Achtziger A. Gollwitzer P. M. Sheeran P. (2008). Implementation intentions and shielding goal striving from unwanted thoughts and feelings. *Pers. Soc. Psychol. Bull.* 34 381–393. 10.1177/0146167207311201 18272806

[B2] Aiken L. S. West S. G. Reno R. R. (1991). *Multiple Regression: Testing and Interpreting Interactions.* New York, NY: Sage.

[B3] Asih D. Teofilus T. Sutrisno T. F. Yoana C. (2020). The effectiveness of social media based on photo and video sharing to-wards online purchase intention. *J. Siasat Bisnis* 24 179–186.

[B4] Barasch A. Diehl K. Silverman J. Zauberman G. (2017a). Photographic memory: the effects of volitional photo taking on memory for visual and auditory aspects of an experience. *Psychol. Sci.* 28 1056–1066. 10.1177/0956797617694868 28650721

[B5] Barasch A. Zauberman G. Diehl K. (2017b). How the intention to share can undermine enjoyment: photo-taking goals and evaluation of experiences. *J. Consum. Res.* 44 1220–1237. 10.1093/jcr/ucx112

[B6] Birtus M. Lăzăroiu G. (2021). The neurobehavioral economics of the COVID-19 pandemic: consumer cognition, perception, sentiment, choice, and decision-making. *Analysis Metaphys.* 20 89–101. 10.22381/am2020216

[B7] Blumer H. (1986). *Symbolic Interactionism: Perspective and Method.* Berkeley, CA: Univ of California Press.

[B8] Byun K.-A. Jones R. P. Wooldridge B. R. (2018). It is not always about brand: design-driven consumers and their self-expression. *J. Retail. Consum. Serv.* 43 296–303. 10.1016/j.jretconser.2018.04.009

[B9] Chae I. Stephen A. T. Bart Y. Yao D. (2017). Spillover effects in seeded word-of-mouth marketing campaigns. *Mark. Sci.* 36 89–104.

[B10] Chu X. Ji S. Wang X. Yu J. Chen Y. Lei L. (2021). Peer phubbing and social networking site addiction: the mediating role of social anxiety and the moderating role of family financial difficulty. *Front. Psychol.* 12:670065. 10.3389/fpsyg.2021.670065 34421727 PMC8374054

[B11] Coary S. Poor M. (2016). How consumer-generated images shape important consumption outcomes in the food domain. *J. Consum. Market.* 33 1–8. 10.1108/JCM-02-2015-1337

[B12] Creusen M. E. H. Gemser G. Candi M. (2018). The influence of experiential augmentation on product evaluation. *Eur. J. Market.* 52 925–945. 10.1108/EJM-04-2016-0220

[B13] Csikszentmihalhi M. (2020). *Finding Flow: The Psychology of Engagement with Everyday Life.* Paris: Hachette UK.

[B14] Dawson J. F. Richter A. W. (2006). Probing three-way interactions in moderated multiple regression: development and application of a slope difference test. *J. Appl. Psychol.* 91 917–926. 10.1037/0021-9010.91.4.917 16834514

[B15] Drugău-Constantin A. (2019). Is consumer cognition reducible to neurophysiological functioning? *Econ. Manag. Financ. Markets* 14 9–14. 10.22381/EMFM14120191

[B16] Duan J. Dholakia R. R. (2017). Posting purchases on social media increases happiness: the mediating roles of purchases’ impact on self and interpersonal relationships. *J. Consum. Market.* 34 404–413. 10.1108/JCM-07-2016-1871

[B17] Duffett R. G. (2015). The influence of Facebook advertising on cognitive attitudes amid Generation Y. *Electron. Commer. Res.* 15 243–267. 10.1007/s10660-015-9177-4

[B18] Felix R. Rauschnabel P. A. Hinsch C. (2017). Elements of strategic social media marketing: a holistic framework. *J. Bus. Res.* 70 118–126. 10.1016/j.jbusres.2016.05.001

[B19] Gao J. Wang J. Bailey A. (2021). How does public recognition affect price sensitivity to green products? The role of self-construal and temporal distance. *Psychol. Market.* 38 1262–1279. 10.1002/mar.21500

[B20] Gilovich T. Kumar A. Jampol L. (2015). A wonderful life: experiential consumption and the pursuit of happiness. *J. Consum. Psychol.* 25 152–165. 10.1016/j.jcps.2014.08.004

[B21] Glass C. R. (2017). Self-expression, social roles, and faculty members’ attitudes towards online teaching. *Innov. High. Educ.* 42 239–252. 10.1007/s10755-016-9379-2

[B22] Godes D. Mayzlin D. (2004). Using online conversations to study word-of-mouth communication. *Mark. Sci.* 23 545–560. 10.1287/mksc.1040.0071 19642375

[B23] Graessley S. Horak J. Kovacova M. Valaskova K. Poliak M. (2019). Consumer attitudes and behaviors in the technology-driven sharing economy: motivations for participating in collaborative consumption. *J. Self Govern. Manag. Econ.* 7 25–30. 10.22381/JSME7120194

[B24] Grewal L. Stephen A. T. Coleman N. V. (2019). When posting about products on social media backfires: the negative effects of consumer identity signaling on product interest. *J. Mark. Res.* 56 197–210. 10.1177/0022243718821960

[B25] Grieve R. Indian M. Witteveen K. Anne Tolan G. Marrington J. (2013). Face-to-face or Facebook: can social connectedness be derived online? *Comput. Hum. Behav.* 29 604–609. 10.1016/j.chb.2012.11.017

[B26] Guo J. Wang X. Wu Y. (2020). Positive emotion bias: role of emotional content from online customer reviews in purchase decisions. *J. Retail. Consum. Serv.* 52 1–11. 10.1016/j.jretconser.2019.101891

[B27] Hayes A. F. (2018). *Introduction to Mediation, Moderation, and Conditional Process Analysis: A Regression-Based Approach* 2nd Edn. New York, NY: Guilford Press.

[B28] Hellmann A. Endrawes M. Mbuki J. (2021). The role of ethnicity in whistleblowing: the case of Kenyan auditors. *Int. J. Audit*. 25 733–750. 10.1111/ijau.12246

[B29] Howard D. J. Gengler C. (2001). Emotional contagion effects on product attitudes. *J. Consum. Res.* 28 189–201. 10.1086/322897

[B30] Jansson A. (2018). Rethinking post-tourism in the age of social media. *Ann. Touris. Res.* 69 101–110. 10.1016/j.annals.2018.01.005

[B31] Kim H. Park K. Schwarz N. (2010). Will this trip really be exciting? The role of incidental emotions in product evaluation. *J. Consum. Res.* 36 983–991. 10.1086/644763

[B32] Krämer N. C. Feurstein M. Kluck J. P. Meier Y. Rother M. Winter S. (2017). Beware of selfies: the impact of photo type on impression formation based on social networking profiles. *Front. Psychol.* 8:188. 10.3389/fpsyg.2017.00188 28261129 PMC5311061

[B33] Lambert N. M. Gwinn A. M. Baumeister R. F. Strachman A. Washburn I. J. Gable S. L. (2013). A boost of positive affect: the perks of sharing positive experiences. *J. Soc. Pers. Relatsh.* 30 24–43. 10.1177/0265407512449400

[B34] Lee G. Lee J. Kwon S. (2010). Use of social-networking sites and subjective well-being: a study in South Korea. *Cyberpsychol. Behav. Soc. Network.* 14 151–155. 10.1089/cyber.2009.0382 20649450

[B35] Lien C. H. Cao Y. (2014). Examining WeChat users’ motivations, trust, attitudes, and positive word-of-mouth: evidence from China. *Comput. Hum. Behav.* 41 104–111. 10.1016/j.chb.2014.08.013

[B36] Liu Y. (2016). *The Research on the Impact of Mobile Music Application Social Experience to the Purchase Intention—The Mediating Effects of Brand Attachment.* Master’s thesis. Guangzhou: Jinan University.

[B37] Loewenstein G. Prelec D. (1992). Anomalies in intertemporal choice: evidence and an interpretation. *Q. J. Econ.* 107 573–597. 10.2307/2118482

[B38] Malik M. S. I. Hussain A. (2017). Helpfulness of product reviews as a function of discrete positive and negative emotions. *Comput. Hum. Behav.* 73 290–302. 10.1016/j.chb.2017.03.053

[B39] Markus H. R. Kitayama S. (1991). Culture and the self: implications for cognition, emotion, and motivation. *Psychol. Rev.* 98 224–253. 10.1037/0033-295X.98.2.224

[B40] Martínez-Ruiz M. P. Gómez-Suárez M. Jiménez-Zarco A. I. Izquierdo-Yusta A. (2017). From consumer experience to affective loyalty: challenges and prospects in the psychology of consumer behavior 3.0. *Front. Psychol.* 8:2224. 10.3389/fpsyg.2017.02224 29312072 PMC5742239

[B41] Meilhan D. (2019). Customer value co-creation behavior in the online platform economy. *J. Self Govern. Manag. Econ.* 7 19–24. 10.22381/JSME7120193

[B42] Mirică C.-O. (2019). The behavioral economics of decision making: explaining consumer choice in terms of neural events. *Econ. Manag. Financ. Markets* 14 16–20. 10.22381/EMFM14120192

[B43] Monga A. B. John D. R. (2008). When does negative brand publicity hurt? The moderating influence of analytic versus holistic thinking. *J. Consum. Psychol.* 18 320–332. 10.1016/j.jcps.2008.09.009

[B44] Naaman M. Boase J. Lai C.-H. (2010). “Is it really about me? message content in social awareness streams,” in *Proceedings of the 2010 ACM conference on Computer supported cooperative work*, (Savannah, Georgia, USA: Association for Computing Machinery).

[B45] Nisbett R. E. Peng K. Choi I. Norenzayan A. (2001). Culture and systems of thought: holistic versus analytic cognition. *Psychol. Rev.* 108 291–310. 10.1037//0033-295X.108.2.291 11381831

[B46] Nowlis S. M. Mandel N. McCabe D. B. (2004). The effect of a delay between choice and consumption on consumption enjoyment. *J. Consum. Res.* 31 502–510. 10.1086/425085

[B47] Ouyang Z. Gursoy D. Sharma B. (2017). Role of trust, emotions and event attachment on residents’ attitudes toward tourism. *Tourism Manage.* 63 426–438. 10.1016/j.tourman.2017.06.026

[B48] Ponsignon F. Derbaix M. (2020). The impact of interactive technologies on the social experience: an empirical study in a cultural tourism context. *Tour. Manag. Perspect.* 35 1–12. 10.1016/j.tmp.2020.100723

[B49] Pop R. Săplăcan Z. Dabija D. C. Alt A. M. (2021). The impact of social media influencers on travel decisions: the role of trust in consumer decision journey. *Curr. Issues Tourism* 25 823–843. 10.1080/13683500.2021.1895729

[B50] Ramanathan S. McGill A. L. (2007). Consuming with others: social influences on moment-to-moment and retrospective evaluations of an experience. *J. Consum. Res.* 34 506–524. 10.1086/520074

[B51] Rifkin J. R. Du K. M. Berger J. (2021). Penny for your preferences: leveraging self-expression to encourage small prosocial gifts. *J. Mark.* 85 204–219. 10.1177/0022242920928064

[B52] Rozenkrants B. Wheeler S. C. Shiv B. (2017). Self-expression cues in product rating distributions: when people prefer polarizing products. *J. Consum. Res.* 44 759–777. 10.1093/jcr/ucx067

[B53] Rydell L. Kucera J. (2021). Cognitive attitudes, behavioral choices, and purchasing habits during the COVID-19 pandemic. *J. Self Govern. Manag. Econ.* 9 35–47. 10.22381/jsme9420213

[B54] Schwarz N. (2011). Feelings-as-information theory. *Handb. Theor. Soc. Psychol.* 1 289–308.

[B55] Shi Y. Luo Y. L. Yang Z. Liu Y. Bao H. (2018). Do narcissists enjoy visiting social networking sites? It depends on how adaptive they are. *Front. Psychol.* 9:1739. 10.3389/fpsyg.2018.01739 30283384 PMC6156359

[B56] Singelis T. M. (1994). The measurement of independent and interdependent self-construals. *Pers. Soc. Psychol. Bull.* 20 580–591. 10.1177/0146167294205014

[B57] Su L. Swanson S. R. (2017). The effect of destination social responsibility on tourist environmentally responsible behavior: compared analysis of first-time and repeat tourists. *Tourism Manage.* 60 308–321. 10.1016/j.tourman.2016.12.011

[B58] Su L. Cheng J. Swanson S. R. (2020a). The impact of tourism activity type on emotion and storytelling: the moderating roles of travel companion presence and relative ability. *Tourism Manage.* 81 1–12. 10.1016/j.tourman.2020.104138

[B59] Su L. Hsu M. K. Boostrom R. E. Jr. (2020b). From recreation to responsibility: increasing environmentally responsible behavior in tourism. *J. Bus. Res.* 109 557–573.

[B60] Su L. Hsu M. K. Marshall K. P. (2014). Understanding the relationship of service fairness, emotions, trust, and tourist behavioral intentions at a city destination in China. *J. Travel Tour. Mark.* 31 1018–1038. 10.1080/10548408.2014.892466

[B61] Su L. Swanson S. R. Chen X. (2016). The effects of perceived service quality on repurchase intentions and subjective well-being of Chinese tourists: the mediating role of relationship quality. *Tourism Manage.* 52 82–95. 10.1016/j.tourman.2015.06.012

[B62] Tamir D. I. Mitchell J. P. (2012). Disclosing information about the self is intrinsically rewarding. *Proc. Natl. Acad. Sci. U.S.A.* 109 8038–8043. 10.1073/pnas.1202129109 22566617 PMC3361411

[B63] Taylor D. G. (2020). Putting the “self” in selfies: how narcissism, envy and self-promotion motivate sharing of travel photos through social media. *J. Travel Tour. Mark.* 37 64–77. 10.1080/10548408.2020.1711847

[B64] Triantafillidou A. Siomkos G. (2014). Consumption experience outcomes: satisfaction, nostalgia intensity, word-of-mouth communication and behavioural intentions. *J. Consum. Market.* 31 526–540. 10.1108/JCM-05-2014-0982

[B65] Walsh G. Shiu E. Hassan L. M. Michaelidou N. Beatty S. E. (2011). Emotions, store-environmental cues, store-choice criteria, and marketing outcomes. *J. Bus. Res.* 64 737–744. 10.1016/j.jbusres.2010.07.008

[B66] Watson D. Clark L. A. (1992). On traits and temperament: general and specific factors of emotional experience and their relation to the five-factor model. *J. Pers.* 60 441–476. 10.1111/j.1467-6494.1992.tb00980.x 1635050

[B67] Watson R. Popescu G. H. (2021). Will the COVID-19 pandemic lead to long-term consumer perceptions, behavioral intentions, and acquisition decisions? *Econ. Manag. Financial Markets* 16 70–83. 10.22381/emfm16420215

[B68] Westbrook R. A. Oliver R. L. (1991). The dimensionality of consumption emotion patterns and consumer satisfaction. *J. Consum. Res.* 18 84–91. 10.1086/209243

[B69] Wilcox K. Stephen A. T. (2013). Are close friends the enemy? Online social networks, self-esteem, and self-control. *J. Consum. Res.* 40 90–103. 10.1086/668794

[B70] Ybarra O. Trafimow D. (1998). How priming the private self or collective self affects the relative weights of attitudes and subjective norms. *Pers. Soc. Psychol. Bull.* 24 362–370. 10.1177/0146167298244003

[B71] Young K. (2011). Social ties, social networks and the Facebook experience. *Int. J. Emerg. Technol. Soc.* 9 20–34.

[B72] Zablocki A. Makri K. Houston M. J. (2019). Emotions within online reviews and their influence on product attitudes in Austria, USA and Thailand. *J. Interact. Mark.* 46 20–39. 10.1016/j.intmar.2019.01.001

[B73] Zell A. L. Moeller L. (2018). Are you happy for me …on Facebook? The potential importance of “likes” and comments. *Comput. Hum. Behav.* 78 26–33. 10.1016/j.chb.2017.08.050

[B74] Zhai S. Sun X. Li J. (2019). Exploring the continuance use intention of mobile APPs based on social experience —An example of NetEase CloudMusic. *J. Mod. Inf.* 39 128–135.

[B75] Zhang Y. Trusov M. Stephen A. T. Jamal Z. (2017). Online shopping and social media: friends or foes? *J. Mark.* 81 24–41. 10.1509/jm.14.0344 11670861

[B76] Zhu J. Jiang L. Dou W. Liang L. (2019). Post, eat, change: the effects of posting food photos on consumers’ dining experiences and brand evaluation. *J. Interact. Mark.* 46 101–112. 10.1016/j.intmar.2018.10.002

[B77] Zhu R. Meyers-Levy J. (2009). The influence of self-view on context effects: how display fixtures can affect product evaluations. *J. Mark. Res.* 46 37–45. 10.1509/jmkr.46.1.37 11670861

[B78] Zollo L. (2021). The consumers’ emotional dog learns to persuade its rational tail: toward a social intuitionist framework of ethical consumption. *J. Bus. Ethics* 168 295–313. 10.1007/s10551-019-04420-4

